# Blood cell for the differentiation of airway inflammatory phenotypes in COPD exacerbations

**DOI:** 10.1186/s12890-020-1086-1

**Published:** 2020-02-24

**Authors:** Jie Gao, Bida Chen, Sifang Wu, Feng Wu

**Affiliations:** Department of Respiratory Medicine, Huizhou third people’s Hospital, Guangzhou Medical College, 1# Xuebei Ave, Huizhou, 516002 Guangdong China

**Keywords:** Sputum, Blood, Eosinophil, Neutrophil, Inflammatory ratios, COPD exacerbations

## Abstract

**Background:**

Measurement of sputum is frequently used to define airway inflammatory subtypes. The venous blood cell is a reliable and simple biomarker, may be used as an alternative procedure to reflect the subtypes. For the aim of verifying the hypothesis that venous blood cell can quantify sputum inflammatory cell to access the airway subtypes in chronic obstructive pulmonary disease of acute exacerbations (AECOPD) and to ascertain the accuracy of the blood cell biomarker.

**Methods:**

This study evaluated 287 patients with COPD exacerbations and all four tests were performed on the same day, which are lung function test, bronchodilator reversibility test, sputum cell analysis and blood routine examination.

**Results:**

There was a correlation between sputum eosinophils and blood eosinophils, blood cells derived ratios. There was a weaker relationship to neutrophils between sputum and blood. Sputum neutrophils had not any association with neutrophil/macrophage ratio (NMR) and eosinophil/lymphocyte ratio (ELR) in blood. Blood eosinophils percentage was predictive for eosinophilic COPD exacerbations with an area under the curve (AUC) of 0.672 (*p* = 0.012). The optimum cutpoint for blood eosinophils percentage was 0.55%. Blood eosinophils absolute count was also predictive sputum eosinophilia at 0.35 × 10^9^/L (AUC = 0.626, *p* = 0.025). ELR, eosinophil/monocyte ratio (EMR) and eosinophil/neutrophil ratio (ENR) in blood were higher in COPD exacerbations with mixed granulocytic and eosinophilic subtypes.

**Conclusion:**

Eosinophils/neutrophils count parameters were relationship between blood and sputum. Eosinophils in blood and the ratios (ENR, EMR and ELR) may be utilized to assess eosinophilic airway inflammation in COPD exacerbations. Due to weak relationship and poor predictive ability, more researches should be required.

## Background

Chronic obstructive pulmonary disease (COPD) is a common, preventable and treatable disease that is characterized by persistent respiratory symptoms and airflow limitation that is due to airway and/or alveolar abnormalities usually caused by significant exposure to noxious particles or gases [1]. Acute exacerbations of COPD (AECOPD) are defined as an acute worsening of respiratory symptoms that result in additional therapy and important events in the management of COPD because they negatively impact health status, rates of hospitalization and readmission, and disease progression [2, 3]. Chronic airway inflammation in COPD exacerbations with increased numbers of inflammatory cell, such as neutrophils, eosinophils, macrophages, and lymphocytes, are response to different parts of the lung and lead to structural changes. Specific airway inflammatory cell types reflected different airway inflammatory phenotypes. Typically, COPD exacerbation is linked to a neutrophilic signature response. The shared pathway probably just represents half of COPD patients. Otherwise, COPD with eosinophilia (sputum eosinophilia > 3%) is present in 10–40% of patients [4, 5]. Eosinophilia COPD shows a favorable response to corticosteroid therapy [6, 7] and Eosinophilic inflammatory airways disease may highlight different genetic, biologic, and pathologic processes with increased all-cause mortality [8].

Four airway inflammatory subgroups were defined by the percentage of eosinophils and neutrophils in sputum. Based on the percentage of eosinophils and neutrophils in sputum, it was to define four airway inflammatory subgroups: eosinophilic, neutrophilic, mixed granulocytic and paucigranulocytic [9]. However, access to this test has a number of limitations, it is unsuitable for point-of-care testing, it requires expertise and it may not be always successful. Due to these reasons, there is a requirement applicable diagnostic tool to predict airway inflammatory phenotypes. Peripheral blood cells may be a potential alternative to quantify sputum inflammatory cells to detect airway inflammatory phenotypes. Recent reports have demonstrated the association between sputum and blood eosinophils in COPD [10, 11]. And the 2018 Global Initiative for Chronic Obstruction Lung Disease (GOLD) published an evidence-based clinical research guideline that blood eosinophils (> 2%) can predict the risk of exacerbations [1]. However, very few studies have addressed the subgroups of airway inflammatory during clinical acute exacerbations.

## Methods

### Participants

This retrospective study was conducted at Huizhou the third people’s Hospital of Guangzhou Medical College between May 2015 and May 2018. All the patients were diagnosed according to the guidelines of GOLD (2018), included airflow obstruction (post-bronchodilator confirm forced expiratory volume in 1 sec (FEV_1_) to forced vital capacity (FVC) ratio [FEV_1_/FVC] < 70%) and an acute worsening of respiratory symptoms that result in additional therapy in a clinical context [1]. They had received a COPD diagnosis at least 1 year before the study. All patients had not used any oral or/and ICS in the previous 8 weeks. We excluded patients if they had pulmonary tuberculosis and interstitial lung disease and lung cancer.

### Ethics statement

The study protocol was approved by the Institutional Review Board of Huizhou the third people’s Hospital, which absolved the need for written informed consent because of the retrospective study. All personal identification data were anonymized and de-identified before analysis.

### Study design

Two hundred eighty-seven patients patients with COPD exacerbations have been participated in the study and all four tests were conducted on the same day, which are spirometry, bronchodilator reversibility, sputum analysis and routine blood.

Spirometry: Spirometry used professional device (MS-pneumo+aps; JAEGER; German) to perform. According to the 2014 guidelines of the China, the quality and criteria of spirometry, which were characteristics of rapid rise in flow/volume curve, duration of expiration more than or equal to 1 sec and visualization of peak expiratory flow (PEF), were required. Repeat at least three times (a variation of no more than 150 ml between two values) and the best was retained [12].

Post-bronchodilator reversibility tests: Inhale 400 μg salbutamol via a metered dose inhaler after baseline and spirometry was repeated after 15–20 min. Three forced expiratory maneuvers were recorded. Post-bronchodilator FEV_1_/FVC < 70% and the negative response (defined as FEV_1_ < 200 ml and FEV_1_ < 12%) were obtained [12].

Sputum samples: Collected lower respiratory sputum portions were dispersed using 0.1% dithiothreitol with water bath (37 °C) and oscillator at 15 min before 300 mesh nylon mesh filter. Total cell counts were centrifuged, smeared and stained (Hematoxylin-Eosin). A differential cell counts were obtained from 400 cells with 400× microscope to identify the phenotypes of airway inflammation. The percentage of sputum eosinophilis ≥2.5% was defined as abnormal [13, 14].

Blood samples: Peripheral blood used Automated Hematology Blood Analyzer (ABX Pentra DF120–1; ABX, France) to measure. The differential white blood cell counts were collected.

Airway inflammatory phenotypes: In 2016, the percentage of sputum eosinophils≥2.5% was identified as airway eosinophilia in China [13]. All the patients were stratified. Eosinophilic AECOPD was defined as sputum eosinophilis ≥2.5%. Neutrophilic AECOPD was defined as sputum neutrophils ≥65%. Patients with increased eosinophils and neutrophils were classified as mixed granulocytic AECOPD. Those with normal levels of both eosinophils and neutrophils were classified as having paucigranulocytic AECOPD.

### Statistical analysis

All data were analyzed using SPSS version 22 (IBM Corporation, Armonk, NY, USA). Categorical variables were presented as frequencies and percentages. Data were reported as median with inter-quartile range for continuous variables. A Kruskal–Wallis test was performed in the different subgroups of patients with COPD exacerbations. The relationship between induced sputum cell counts and peripheral blood cell counts was assessed with the Spearman’s rank correlation coefficient. Correlation between tests was performed by constructing receiver operating characteristic (ROC) curve. The optimal cutoff value was determined from the highest sum of sensitivity and specificity. A *p* value < 0.05 was considered statistically significant.

## Results

### Characteristics of the patients

We evaluated 287 patients with COPD exacerbations who underwent lung function, sputum cells and blood samples. Patients demographic information are demonstrated in Table 1. The median age of patients was 76 years. After a post-bronchodilator, the median FEV1/FVC% was 49.8% and FEV1% predicted was 46.8%.

**Table 1:** Patient demographic characteristics

Parameters	All patients (*N* = 287)
Mean age, years	75(68–80)
Males, n (%)	182(63.41)
BMI, kg/m 2	20.7(18.3–23.42)
FVC (L)	2.11 (1.66–2.68)
FEV1(L)	0.99 (0.68–1.44)
FEV1% predicted	46.8 (31–63.85)
FEV1/FVC (%)	49.8 (39.12–58.9)
Smokers, n (%)	156(54.36)
Sputum eosinophils %	1(0–2.79)
Sputum neutrophils %	92.56(82.35–96.11)
Sputum lymphocytes %	1.5(0.5–3)
Sputum macrophages %	2.22(0.83–8.17)

Note: N refers to the total population; n refers to the sub-group population; *BMI* Body mass index, *FVC* Forced vital capacity, *FEV*1 Forced expiratory volume in 1 s;

Values are expressed as median (inter-quartile range)

### Blood cells and inflammatory cell ratios

An adequate sputum sample was obtained (oral squamous cell counts less than 10% in each sample was deemed adequate for further analysis). COPD exacerbations airway inflammatory phenotypes were defined by using sputum eosinophils and neutrophils. Patients were classified into four groups: neutrophilic AECOPD (*n* = 152), eosinophilic AECOPD (*n* = 8), mixed granulocytic AECOPD (*n* = 104) and paucigranulocytic AECOPD (*n* = 23) (Table 2).

**Table 2:** Blood cells and inflammatory cell ratios according to sputum inflammatory phenotype

	All	Neutrophilic (*n* = 152)	Eosinophilic (*n* = 8)	Mixed granulocytic (*n* = 104)	Paucigranulocytic (*n* = 23)	*P*-value
White blood cell (× 10^9^/L)	7.6 (5.9–10.5)	8.2 (6.2–11)	6.15 (5.05–6.58)	6.4 (5.5–8.6)#	7.95 (5.75–11.5)	< 0.001
Neutrophils (× 10^9^/L)	5.5 (4.18–8.2)	6.1 (4.4–8.9)	4 (2.83–4.3)	4.7 (3.68–6.25)#	5.85 (4.25–8.95)	< 0.001
Eosinophils (× 10^9^/L)	0.1 (0–0.1)	0 (0–0.1)	0.25 (0.13–0.3)*#	0.1 (0–0.2)*#	0 (0–0.1)	< 0.001
Leukocytes (×10^9^/L)	1.2 (0.8–1.8)	1.2 (0.7–1.7)	1.3 (1.05–2.3)	1.3 (1–1.73)#	1.3 (1.05–1.9)	=0.022
Monocytes (×10^9^/L)	0.4 (0.3–0.6)	0.4 (0.3–0.7)	0.45 (0.4–0.73)	0.4 (0.3–0.6)	0.4 (0.2–0.6)	=0.079
Neutrophils %	76.3 (67.28–83.53)	78.6 (70.45–84.75)	63.95 (51–70.15)	70.9 (62.68–78.55)#	73.9 (63.63–84.38)	< 0.001
Eosinophils %	0.5 (0.2–1.73)	0.3 (0.2–0.95)	4.3 (1.88–6.5)*#	1.6 (0.4–3.03)*#	0.3 (0.18–1.7)	< 0.001
Leukocytes %	16.2 (10.78–23.6)	14.7 (9.45–21.6)	25.7 (19.3–31.73)	19.7 (14.1–26.63)#	18.35 (11.1–27.7)	< 0.001
Monocytes %	5.4 (3.8–7.3)	5.4 (3.7–7.45)	7.2 (5.93–9.88)	5.6 (4.5–7.13)	3.85 (3.33–7)	=0.091
Blood NLR	4.72 (2.8–7.68)	5.38 (3.2–9)	2.65 (1.63–3.68)	3.6 (2.45–5.63)#	3.81 (2.22–7.89)	< 0.001
Blood ELR	0.03 (0–0.1)	0 (0–0.08)	0.14 (0.24–0.08)*#	0.09 (0–0.16)*#	0 (0–0.07)	< 0.001
Blood ENR	0.01 (0–0.03)	0 (0–0.02)	0.06 (0.03–0.12)*#	0.02 (0–0.06)*#	0 (0–0.02)	< 0.001
Blood EMR	0.07 (0–0.33)	0 (0–0.21)	0.45 (0.24–0.73)*#	0.25 (0–0.5)*#	0 (0–0.31)	< 0.001
Blood NMR	13.27 (9.33–20.28)	14 (9.76–21.93)	8.3 (4.9–10.44)	12 (8.92–16.64)	18.5 (9.7–26)	=0.004
Blood MLR	0.33 (0.22–0.5)	0.37 (0.25–0.6)	0.33 (0.29–0.37)	0.29 (0.2–0.42)#	0.25 (0.15–0.41)#	< 0.001

*NLR* Neutrophil/lymphocyte ratio, *ELR* Eosinophil/lymphocyte ratio, *ENR* Eosinophil/neutrophil ratio, *EMR* Eosinophil/macrophage ratio, *NMR* Neutrophil/macrophage ratio, *MLR* Macrophage/lymphocyte ratio;

According to Bonferroni principle, *P < 0.008, paucigranulocytic AECOPD is used as the comparator; #*P* < 0.008, Neutrophilic AECOPD is used as the comparator

Data are presented as median (inter-quartile range)

**Fig. 1:**
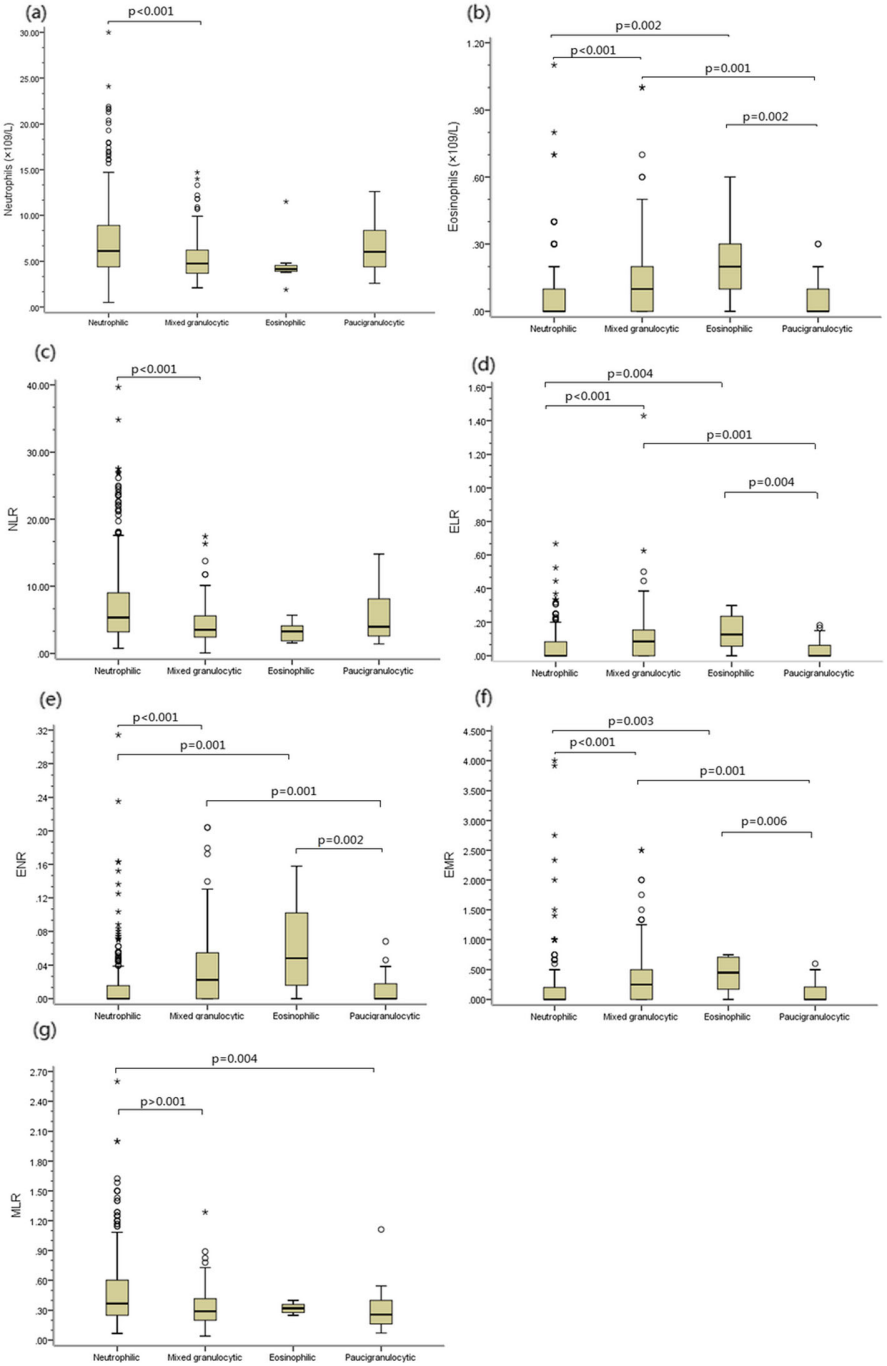
Box plot comparing. Notes: Four inflammatory phenotypes were classified according to the percentage of sputum eosinophils (≥2.5%) and neutrophils (≥65%). The box plot shows the median and interquartile values. (**a)** the absolute blood neutrophil count; (**b)** the absolute blood eosinophil count; (**c**) the neutrophil/lymphocyte ratio (NLR); (**d**) the eosinophil/lymphocyte ratio (ELR); (**e**) the eosinophil/neutrophil ratio (ENR); (**f)** the eosinophil/macrophage ratio (EMR); (**g**) the macrophage/lymphocyte ratio (MLR)

Peripheral blood cell parameters and blood inflammatory cell ratios are presented in Table 2. Eosinophilic and mixed granulocytic AECOPD both showed an increase in the number and percentage of blood eosinophils (Table 2, Fig. 1b). Patients with neutrophilic AECOPD had a higher total white blood cell counts and neutrophil absolute/ percentage compared with mixed granulocytic AECOPD, but a reduction in the number and proportion of leukocyte compared with mixed granulocytic phenotypes (Table 2, Fig. 1a). Monocyte in peripheral blood percentage and absolute count were similar across phenotypes.

The blood eosinophil/lymphocyte ratio (ELR), eosinophil/neutrophil ratio (ENR) and eosinophil/monocyte ratio (EMR) were significantly higher in eosinophilic and mixed granulocytic group compared with other groups (Table 2, Fig. 1d-f). The blood neutrophil/lymphocyte ratio (NLR) was increased to neutrophilic compared with mixed granulocytic phenotype (Table 2, Fig. 1c). The blood macrophage/lymphocyte ratio (MLR) was different from neutrophilic compared with paucigranulocytic phenotype (Table 2, Fig. 1g). The blood neutrophil/macrophage ratio (NMR) was not different from groups (Table 2).

### Associations between sputum cells, blood cells and inflammatory cell ratios

**Fig. 2:**
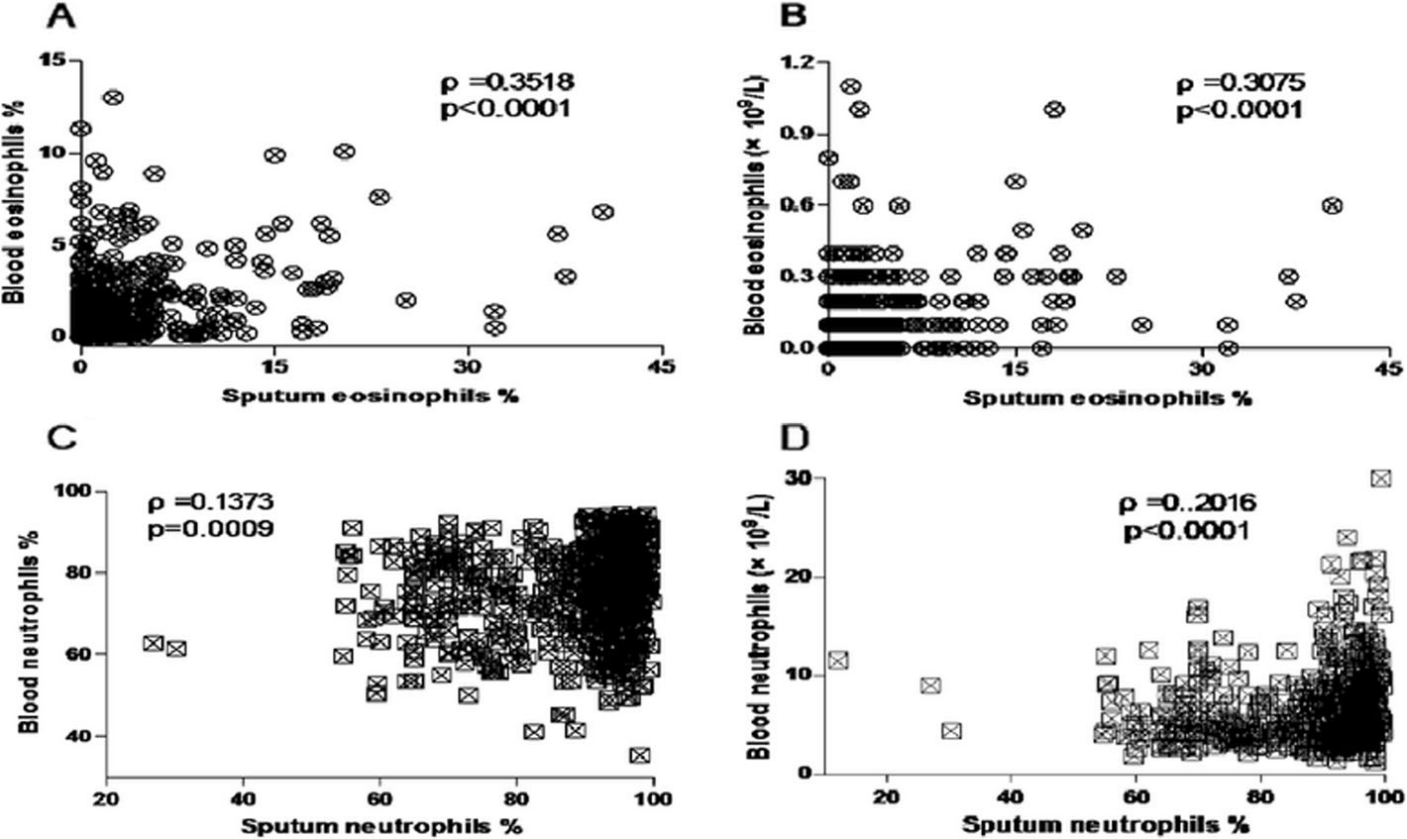
scatter plots for correlations between eosinophil and neutrophil in sputum and blood. (**a**) Correlation between the percentage of sputum eosinophils and the percentage of blood eosinophils. (**b**) Correlation between the percentage of sputum eosinophils and the absolute blood eosinophil count (× 10^9^/L). (**c**) Correlation between the percentage of sputum neutrophils and the percentage of blood neutrophils. (**d**) Correlation between the percentage of sputum neutrophils and the absolute blood neutrophils count (× 10^9^/L)

There was a significant positive relationship between the percentage of sputum eosinophils and blood eosinophil absolute, blood eosinophil percentage (*ρ* = 0.3075, *p* < 0.0001; *ρ* = 0.3581, *p* < 0.0001; respectively) (Fig. 2a-b). The blood inflammatory cell ratios included not only ELR, ENR, EMR (*ρ* = 0.2793, *p* < 0.0001; *ρ* = 0.348, *p* < 0.0001; *ρ* = 0.3211, *p* < 0.0001; respectively), but also NLR, NMR, MLR (*ρ* = − 0.2882, *p* < 0.0001; *ρ* = − 0.1023, *p* = 0.0133; *ρ* = − 0.2402, *p* < 0.0001; respectively), correlated reasonably well with the percentage of sputum eosinophils. A weaker correlations were between the percentage of sputum neutrophils and blood neutrophil percentage, blood neutrophil absolute (*ρ* = 0.1373, *p* = 0.0009; *ρ* = 0.2016, *p* < 0.0001; respectively) (Fig. 2c-d), blood NLR (*ρ* = 0.1479, *p* = 0.0003), blood MLR (*ρ* = 0.1926, p < 0.0001), blood ENR (*ρ* = − 0.1113, *p* = 0.007) and blood EMR (*ρ* = − 0.1009; *p* = 0.0146). There was no significant relationship between the percentage of sputum neutrophils and blood ELR, blood NMR.

**Fig. 3:**
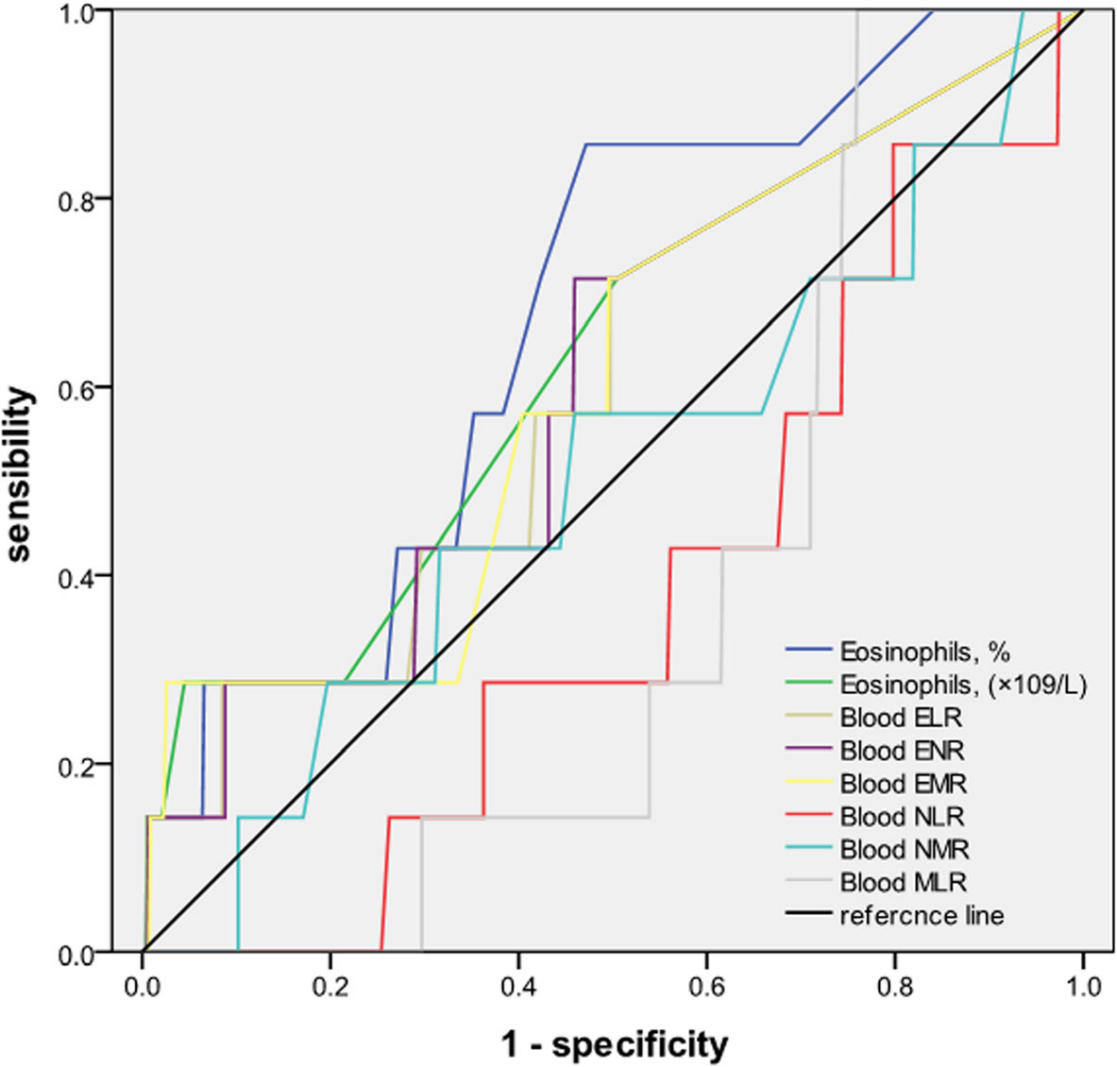
ROC curve analyses of blood parameters for predicting sputum eosinophilia (≥ 2.5%)

The receiver operating characteristic (ROC) curve analysis identified blood eosinophil percentage and absolute blood eosinophil count as the best predictor for airway inflammatory eosinophilia, with an area under the curve (AUC) of 0.672 (*p* = 0.012) and 0.626 (*p* = 0.025), respectively. The optimum cut-point for blood eosinophil percentage was 0.55% (sensitivity was 85.7%, specificity was 52.8%; respectively) and blood eosinophil absolute was 0.35 × 10^9^/L (sensitivity was 71.4%, specificity was 49.6%; respectively), Blood eosinophil ratios, such as ELR, ENR, EMR, were also predictive of sputum eosinophilia with AUCs of 0.601 (*p* = 0.036), 0.603 (*p* = 0.035) and 0.604 (*p* = 0.034), respectively. A summary of sensitivities and specificities of optimal cutoff points of blood eosinophil ratios were provided in the Table 3. Blood NLR, blood NMR and blood MLR were not as efficient as blood eosinophil ratios to detect eosinophilic phenotype (AUC = 0.375, *p* = 0.255; AUC = 0.503, *p* = 0.978, AUC = 0.374, p = 0. 252), although these blood ratios were correlated with the percentage of sputum eosinophils (Table 3, Fig. 3). Neutrophilic phenotype could not be detected by the percentage of blood neutrophils, the absolute blood neutrophil count and the blood inflammatory cell ratios, the ROC curve of these parameters did not show useful values.

**Table 3:** ROC curve analyses of blood parameters for predicting airway inflammatory phenotype

	AUC	*P*-value	95% Confidence interval		Cutoff point	Sensitivity %	Specificity %
			Lower boundary	Upper boundary			
Predicting eosinophilic AECOPD (the percentage of sputum eosinophils ≥2.5%)							
Eosinophils (×10^9^/L)	0.626	0.025	0.407	0.844	0.35	71.4	49.6
Eosinophils %	0.672	0.012	0.494	0.849	0.55	85.7	52.8
Blood ELR	0.601	0.036	0.389	0.813	0.03	71.4	50.3
Blood ENR	0.603	0.035	0.392	0.814	0.01	71.4	54.1
Blood EMR	0.604	0.034	0.387	0.822	0.07	71.4	50.4
Blood NLR	0.375	0.255	0.201	0.549			
Blood NMR	0.503	0.978	0.283	0.723			
Blood MLR	0.374	0.252	0.256	0.492

## Discussion

The study, which evaluated the ability of peripheral blood cells and inflammatory cell ratios for detecting the airway inflammatory phenotypes in COPD exacerbations, had some findings. First, we have shown that eosinophil and neutrophil parameters were correlation between sputum and blood. Second, peripheral blood eosinophil (absolute and percentage) and derived ratios (ELR, ENR and EMR) were poor to distinguish patients with sputum eosinophilia, but significant. Third, white blood cell (× 10^9^/L), blood neutrophil (absolute and percentage) and its ratios (NLR and NMR) may not predict neutrophilic AECOPD. We have also found that blood neutrophil (absolute and percentage) and NLR are decreased in mixed granulocytic AECOPD compared with neutrophilic AECOPD, which may reflect systemic inflammation, but not enough to become clinically useful. COPD exacerbations are complex events mainly relationship to morbidity and mortality, which airway inflammation may play an important role [15]. Most of the events have been observed because of respiratory viral and bacterial infection with increased numbers of specific airway inflammatory cell types [16, 17]. During the process of airway inflammation, numbers of macrophages increased in peripheral airways together with activated neutrophils and CD8 + T-lymphocytes that include interleukin (IL)-8, tumor necrosis factor-alpha (TNF-α), among others (Tc1, Th1, Th17 and ILC3), release multiple inflammatory mediators and play a predominant role in the case of COPD exacerbations. There may be also feasible increases in eosinophils, Th2 or ILC2 cells, especially where there is clinical overlap with asthma. In pure COPD approximately, 30% of patients have an eosinophilic component to their airway inflammation. Moreover, blood eosinophils may fall to very low levels (transient eosinopenia) during sepsis and many severe exacerbations of COPD. All of these inflammatory cells, together with epithelial cells and other structural cells could be recruited to induce the destruction of lung parenchyma tissue (resulting in emphysema) and disruption of normal repair and defense mechanisms (resulting in small airway fibrosis) [1, 18]. These changes contribute to increased dyspnea, cough and wheeze that is symptom of an exacerbation. Other symptoms include increased sputum purulent and volume, together with increasing disease severity.

The different inflammatory phenotypes are also clinically relevant due to potential differences in the response to therapeutic interventions. In one study of COPD exacerbation phenotypes, Bafadhel et al. identified distinct subtypes that were in contact with bacterial or viral infection or sputum eosinophilia and were associated with 55, 29, and 28% of exacerbations, respectively. These were clinically indistinguishable but could be identified by phenotype-specific biomarkers of sputum IL-1β, serum CXCL10 and peripheral eosinophils [19]. Stable state bacterial colonization and sputum eosinophilia predicted bacterial exacerbations and eosinophilic exacerbations, respectively. In contrast, viral exacerbations were not related to stable state phenotype but rather represented the acquisition of a novel pathogen. Bacterial and eosinophilic exacerbations rarely co-existed. Bacterial infection is more likely to be associated with eosinopenia [19]. There are some previous studies to support the concept that eosinophils or/and neutrophils are increased from the airway and blood in a significant proportion of subjects with COPD exacerbations [19, 20]. Sputum eosinophilia has been related to viral infection [16]. It has been suggested that exacerbations associated with an increase in sputum or blood eosinophils may be more responsive to systemic steroids [21]. The effect of treatment is different in eosinophilic airway inflammation of COPD from those without. Therefore, identification of airway inflammatory phenotypes will be of great in understanding the disease and in the management of COPD exacerbations.

Measurement of sputum is not infrequently used to determine the airway inflammatory phenotype, including in our study. Patients can be divided into different phenotypes according to the type of inflammatory cells. Blood cells are simple and accessible biomarkers to obtain in clinical practice. Previous literatures suggested that blood eosinophils may represent a useful surrogate measure of sputum eosinophils in COPD [10, 21], although blood eosinophils appear to a moderate relationship with sputum eosinophils in asthma [22]. According to the 2014 guidelines of China, Eosinophilic airway inflammation was defined as the percentage of sputum eosinophils ≥2.5%, neutrophilic airway inflammation was defined as the percentage of sputum neutrophils ≥65% [13]. Our study has provided a positive relationship between sputum eosinophilia and eosinophils (whether absolute eosinophil counts or percentages) in peripheral blood and presented blood eosinophils absolute count (0.35 × 10^9^/L) may help identify sputum eosinophilia (AUC 0.626, sensitivity =71.4%, specificity =49.6%) in COPD exacerbations, the correlation and ROC analyses demonstrated a relatively poor, although significant relationship for blood eosinophils to predict sputum eosinophilia. The result is similar to Soter et al. data optimal cutoff point (0.3 × 10^9^/L) in COPD exacerbations, but the sensitivity and specificity are different (sensitivity =60%, specificity =76%) [23]. Hastie et al. had reported that blood eosinophils were a weak to predict sputum eosinophils (AUC = 0.64, *p* < 0.0001) and the correlation was poor (r = 0.178, *p* < 0.001) in eosinophils between sputum and blood [24]. Bafadhel’s study showed a much stronger relation between sputum eosinophilia and blood eosinophils during exacerbations with an AUC of 0.85 (95% CI, 0.78–0.93). A cutoff of 2% peripheral blood eosinophils had a sensitivity of 90% and specificity of 60% for identifying a sputum eosinophilia of greater than 3% at exacerbation [19]. Our previous research had revealed that peripheral percentage blood eosinophil (> 0.65%) [11] can be a very good biomarker of sputum eosinophilic airway inflammation in COPD exacerbations (AUC 0.729, sensitivity =74.2%, specificity =61.4%), the optimal cut offs (0.65%) are similar to the result (> 0.55%) in this study. Blood eosinophil ratios, such as ENR, ELR and EMR, also seemingly predicted sputum eosinophilia. These results had demonstrated significantly in statistics. However, larger studies of comprehensively phenotyped COPD patients lacked robust sputum eosinophils data and sputum eosinophilia was defined different from 1 to 3%. Thus it is uncertain whether peripheral eosinophils accurately predict sputum eosinophilia in COPD exacerbations.

RCT of blood eosinophil directed Prednisolone therapy for COPD exacerbations, only patients with blood eosinophils > 2% showed benefit [21]. Other large studies have shown a clear relation between blood eosinophil level and response to ICS [25–27]. The 2018 Global Initiative for GOLD published that blood eosinophils may be biomarkers of exacerbation risk with a history exacerbation and can predict effect of ICS in patients on exacerbation prevention [1]. The guidelines include blood eosinophil as a biomarker to guide ICS treatment. However this recommendation is based on stable blood eosinophil counts in the large studies supporting this approach. It would have been interesting to reassess this population during the stable state. Whilst this was not done, in light of the above we acknowledge that the inflammatory state during COPD exacerbations may differ from stable state, and whilst this may influence management of the exacerbation (larger studies are required), it should not influence long term ICS therapy.

This finding is also reported that blood neutrophils have a relationship to sputum neutrophils, the strength of the correlation was weak and ROC analysis displayed poor results. A study of COPD reported that there was no association of neutrophils in sputum and blood. There was a consensus with previous studies of Hastie et al. [28] and Zhang XY et al. [29] in asthma. As blood neutrophils had a weak correlation with sputum neutrophils, further studies should use in order to evaluate this relationship. Our study showed that neutrophilic AECOPD had a higher NLR, which combines neutrophils (marker of innate inflammation) and lymphocytes (marker of allergic inflammation) as a ratio. A high blood NLR has reported to associate with poor clinical prognosis in various chronic diseases.

### Limitation of the study

Our study reported on a clinical study with 287 patients with COPD on exacerbations to investigate the relationship between inflammatory markers measured in sputum and peripheral blood. Eosinophil measurements were most highly correlated, and neutrophil measurements also showed correlation between sputum and blood. However, overall the association between sputum and blood measurements appeared weak, with an AUC for the prediction of sputum eosinophilia from blood eosinophil percentage of only 0.67 and blood eosinophil absolute of only 0.63. While these correlations might show statistical significance, none of these appear to convince and potentially clinically relevant judged by the scatter plots. Also, the low AUC values support that prediction of sputum measurements of blood measurements is very unreliable and, very likely, not helpful to inform clinical decision making. Statistically significant does not always imply clinical significance, but the level of association needs to be considered, as well.

Another major limitation of the study was the cause of the acute exacerbation. This was usually due to infections, and depending on the infection, changed in peripheral blood cells might be visible that have nothing to do with the COPD. Therefore, the weak correlation between the peripheral blood and sputum values could be an indication for the infections, without any relevance to COPD.

## Conclusions

This study provides evidence that eosinophil/neutrophil parameters were relationship between sputum and blood. Blood eosinophils and its derived ratios (ELR, ENR and EMR) may be utilized to detect airway eosinophilia in AECOPD, but blood neutrophil (absolute and percentage) and its ratios (NLR and NMR) may not. Take into account these weak correlations and poor prediction values additional investigations for differentiating phenotypes would be required.

## Data Availability

The datasets used and/or analysed during the current study are available from the corresponding author on reasonable request.

## Abbreviations

FEV1: Forced expiratory volume in 1 s
FVC: Forced vital capacity
GOLD: Chronic obstruction lung disease
MEF: Maximal expiratory flow
MMEF: Maximum mid-expiratory flow
pef: Peak expiratory flow
